# Establishing Reference Values for Thyroid Vascularity Using Ultra-Micro Angiography (UMA) Ultrasound Technology

**DOI:** 10.3390/diagnostics15040471

**Published:** 2025-02-14

**Authors:** Luciana Moisa-Luca, Andreea Bena, Stefania Bunceanu, Dana Stoian

**Affiliations:** 1 Department of Doctoral Studies, “Victor Babeş” University of Medicine and Pharmacy, 300041 Timişoara, Romania; luciana.luca@umft.ro (L.M.-L.); bunceanu.stefania@gmail.com (S.B.); 2 Center for Molecular Research in Nephrology and Vascular Disease, “Victor Babeş” University of Medicine and Pharmacy, 300041 Timişoara, Romania; stoian.dana@umft.ro; 3 Discipline of Endocrinology, Second Department of Internal Medicine, “Victor Babeş” University of Medicine and Pharmacy, 300041 Timişoara, Romania

**Keywords:** microvascular, Doppler ultrasound, color pixel percentage (CPP), thyroid ultrasound

## Abstract

**Background/Objectives:** Ultra-Micro Angiography (UMA) is an advanced Doppler technique designed to improve the visualization of slow blood flow in small vessels. The Subtraction UMA (sUMA) setting enhances these features by removing background tissue interference, allowing for more precise assessments of microvascularity. This study aims to establish reference values for thyroid vascularity using sUMA technology, providing a foundation for future research in thyroid pathology. **Methods:** This prospective, single-center study included 106 healthy participants with no evidence of thyroid disease based on biochemical and ultrasound evaluations. All participants underwent multiparametric ultrasound, followed by sUMA to assess thyroid vascularity. The quantitative sUMA measurements were performed using the color pixel percentage (CPP), and three measurements were taken in each thyroid lobe. The median CPP values were calculated and analyzed. Statistical analysis was conducted to evaluate intraobserver reliability and to examine correlations between CPP values and demographic characteristics. **Results:** The study cohort had a mean age of 41.2 ± 16.3 years, with a predominance of women (82%). CPP sUMA measurements demonstrated excellent feasibility (100%) and intraobserver reliability, with an intraclass correlation coefficient of 0.905 for the right thyroid lobe and 0.897 for the left lobe. The median CPP for the right and left lobes was 26.5% and 27.1%, respectively, with no significant difference between lobes (*p* = 0.8799). **Conclusions:** sUMA technology is a reliable and reproducible method for evaluating thyroid microvascularity in healthy individuals. These reference values provide a foundation for future studies investigating thyroid pathology, potentially enhancing the accuracy of diagnostic assessments in clinical practice.

## 1. Introduction

Ultrasound (US) assessment of the thyroid parenchyma is essential for distinguishing between normal thyroid tissue and asymptomatic or mild diffuse thyroid diseases (DTDs). Ultrasound features associated with DTDs include altered parenchymal echogenicity, coarse parenchymal echotexture, increased anteroposterior diameter, lobulated glandular margins, and abnormal parenchymal vascularity with high sensitivity (87.7%) and specificity (92.1%) [1,2].

Evaluating thyroid vascularity is valuable in distinguishing the causes of diffuse thyroid disease (DTD) and thyrotoxicosis, as it correlates with the gland’s functional status. Both low and high levels of TSH can alter thyroid blood flow [3]. High levels of TSH stimulate the thyroid gland, increasing blood supply in the early phase of Hashimoto thyroiditis (HT) and untreated hypothyroidism [4,5]. However, in later stages of HT, blood supply decreases because of thyroid follicle destruction and fibrosis [5,6,7].

Color Doppler (CD) is crucial for distinguishing GD from other causes of thyrotoxicosis. It has a high sensitivity of 88.9% and specificity of 87.5% when compared to thyroid scintigraphy [8]. A qualitative assessment of thyroid parenchymal vascularization can be performed using the Schultz scale, which demonstrates hypervascularization of thyroid parenchyma in GD. This phenomenon is closely related to thyroid stimulation by TSH-receptor antibodies and a high level of FT4 [4,5,7,9]. Furthermore, a quantitative evaluation can be obtained by measuring the peak systolic velocity (PSV) in the thyroid artery. PSV values are significantly higher in patients with GD with a cutoff of 40–50 cm/s and a mean value of 42.4 cm/s in differentiating GD from thyroiditis, with excellent diagnostic accuracy [10,11].

The primary limitation of the Doppler method is the presence of motion artifacts (clutter), as the signal is captured both from blood flow and from the movement of surrounding tissues. Applying a clutter rejection filter helps eliminate these artifacts but can result in the loss of Doppler signals from low-velocity small vessels [12,13]. This disadvantage can be overcome by a new wall-filtering algorithm that can separate low-flow signals from clutter for a better assessment of small blood vessels and their distribution in the tissue [14].

Ultra-Micro Angiography (UMA) is an advanced Doppler technique designed to improve the visualization and identification of slow-flood blood vessels using high-quality ultrasound signals through the plane wave and divergent waves, enabling a faster sampling rate and consequently enhancing sensitivity. Additionally, a wall-filtering algorithm allows for the precise differentiation between low-speed blood flow and low-speed tissue movement, providing an accurate visualization of the micro-vessel [15]. UMA has three types of settings, color UMA (cUMA) for the assessment of the velocity and direction of the blood flow, power UMA (pUMA) focused on the power intensity of the blood flow in small vessels, and Subtraction UMA (sUMA) which enhances the features obtained from pUMA by remoting the signals from the background tissue that may interfere with the detection of blood flow [15,16].

The aim of our study is to establish reference values for thyroid vascularity using sUMA technology. By focusing on the power intensity of blood flow in small vessels and minimizing background tissue interference, these values will provide a useful reference for future research in thyroid pathology, supporting more accurate assessments in clinical practice.

## 2. Materials and Methods

### 2.1. Study Cohort

**Figure 1:**
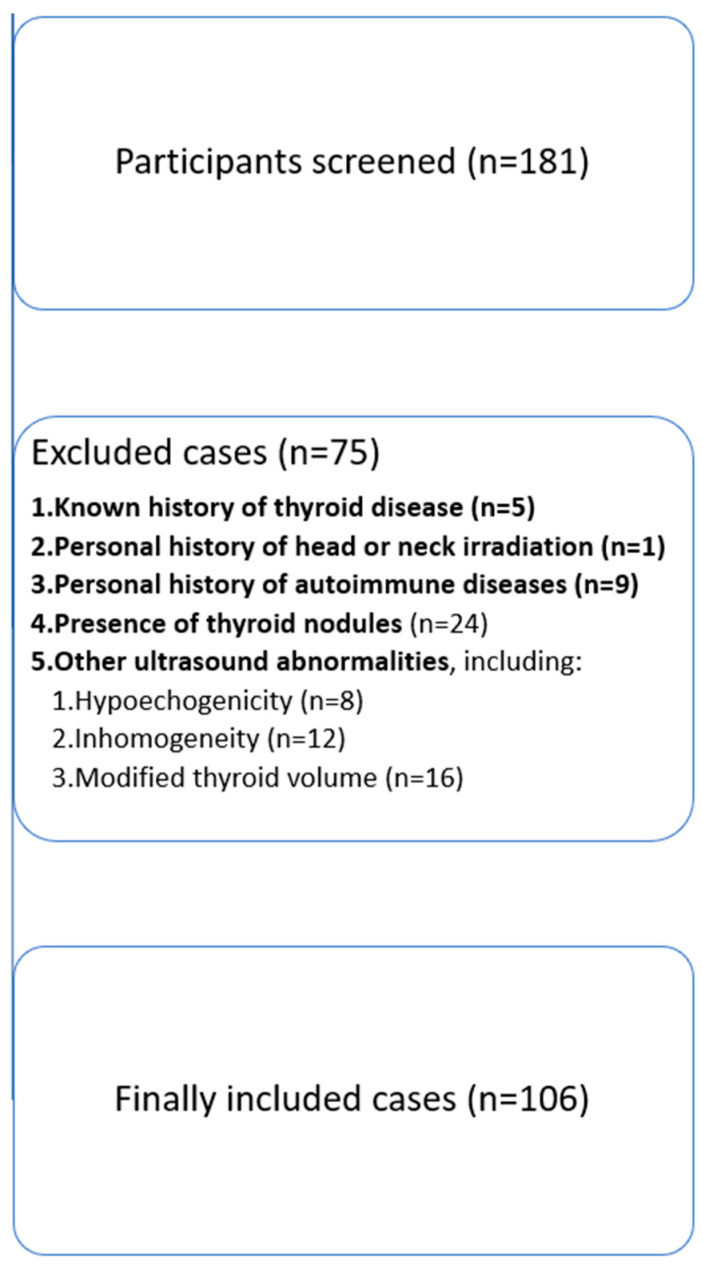
Participant selection flowchart for the study on establishing reference values for thyroid vascularity using sUMA.

A prospective, single-center study was conducted over 6 months (January–June 2024). A total of 106 consecutive participants (Figure 1) that presented in our endocrine ultrasound unit for screening purposes were enrolled, with no evidence of thyroid disease (biochemical—normal thyroid stimulating hormone [TSH] levels; anamnestic—no personal history of thyroid disease diagnosis; and upon current ultrasound examination—a normal thyroid appearance). Exclusion criteria included individuals with a known history of thyroid disease, a personal history of head or neck irradiation, a personal history of autoimmune diseases, or presence of thyroid nodules or other ultrasound abnormalities (hypoechogenicity, inhomogeneity, modified volume). All participants were residents of Timis County, Romania, an area recognized as iodine-sufficient [17].

### 2.2. Ultrasound Examination

For all cases, a multiparametric ultrasound assessment was carried out by the same operator (A.B.), with more than 5 years of practice in thyroid ultrasound. Each subject was evaluated firstly by B-mode ultrasound, immediately followed by sUMA. The patient was positioned supine with the neck hyperextended, and coupling gel was applied between on the skin. All ultrasound examinations were performed using the same machine (Mindray Resona R9, Mindray Bio-Medical Electronics Co., Ltd., Shenzhen, China) and a 3–13 mHz probe (L15-3WU) set on 11 mHz frequency. The thyroid volume was calculated measuring three dimensions in two incidences for each lobe. The presence of nodules was evaluated as well as the echogenicity of the parenchyma. The cases with normal B-mode parameters were subsequently evaluated by sUMA. We selected sUMA for the evaluation of thyroid microvascularity due to its enhanced capability to isolate blood flow signals from background tissue, providing a clearer and more precise visualization of microvascular structures. Unlike other UMA settings, sUMA refines the power intensity signals from pUMA, removing interference from surrounding tissues that may obscure the detection of blood flow. This feature is particularly advantageous for assessing the subtle vascular patterns within normal thyroid tissues, offering a reliable baseline for future comparative studies in pathological settings, where accurate detection of microvascular changes is critical.

**Figure 2:**
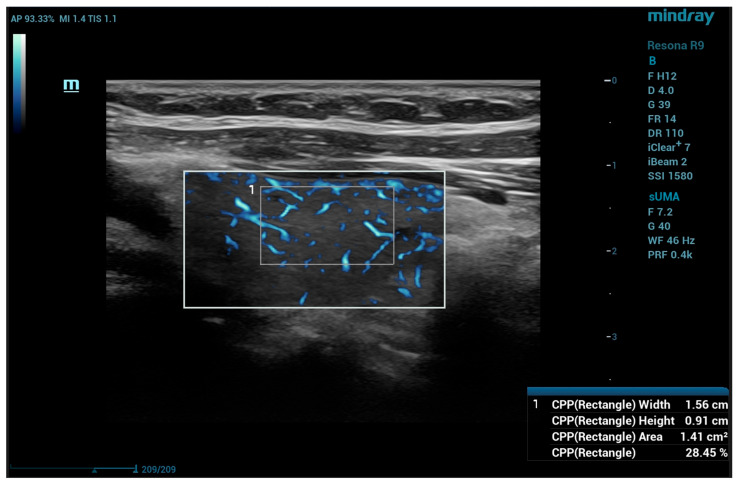
Thyroid microvascularity using UMA technology: quantitative CPP measurement, in a patient without thyroid pathology; CPP—color pixel percentage; UMA—Ultra-Micro Angiography.

The same probe was used for sUMA measurement. In longitudinal view in B-mode, the area with the thickest portion of each lobe was selected and UMA (subset sUMA) was activated. The region of interest (ROI) box size was selected so to ensure the covering of most of the lobe height, starting superficial to the anterior thyroid capsule, with a lateral size of the ROI of a maximum 75% of the field of view. The gain was set to 40 in all cases; the wall filter frequency could be adjusted so order to reduce visible motion artifacts. Before acquiring the images for quantitative measurements, the patient was asked to hold still, with the probe also held still for 5–10 s in order to reduce artifacts caused by motion; if artifacts persisted, the patient was asked to hold their breath until stable images were obtained. The quantitative measurements were performed using the color pixel percentage (CPP) and the rectangle CPP measurement, covering a 0.8–1 per 1.5–2 cm box inside the lobe, adjusted to individual anatomical variants, avoiding areas with visible artifacts. The depth and CPP were registered for three measurements in each lobe (Figure 2). The median value of the three measurements was calculated in each case and the IQR.

### 2.3. Statistical Analysis

Statistical analysis was performed using MedCalc V19.4 (MedCalc Software Ltd., Ostend, Belgium). Descriptive statistics were utilized to summarize clinical, demographic, anthropometric, and ultrasound findings. The distribution of continuous variables was examined using the Kolmogorov–Smirnov test to determine normality. For variables following a normal distribution, results were reported as mean ± standard deviation, whereas non-normally distributed variables were represented by median and interquartile range. Categorical variables were described as percentages and visualized with bar charts or pie charts where applicable. To compare two independent groups with non-normally distributed variables, the Mann–Whitney U test was employed. For multiple group comparisons, an analysis of variance (ANOVA) was conducted for normally distributed variables to detect any significant differences among groups. If the ANOVA revealed significant results, post hoc tests were applied to identify specific group differences. Feasibility and interobserver reliability were assessed to ensure the consistency and reliability of CPP sUMA measurements. Feasibility was defined as the probability of obtaining valid measurements, while interobserver reliability was evaluated using the intraclass correlation coefficient (ICC) for measurements across different observers. When assessing correlations, Pearson’s correlation coefficient was calculated for variables with normal distribution, while Spearman’s rank correlation coefficient was used for data that did not meet normality assumptions. In addition, box plots were generated to visually compare the spread and central tendency of numerical variables across groups, highlighting potential outliers and aiding in the interpretation of distribution characteristics. For all inferential tests, 95% confidence intervals (CIs) were calculated, and statistical significance was established at a *p*-value threshold of less than 0.05.

## 3. Results

### 3.1. Baseline Profile of Study Participants

According to BMI, the participants had a mean value of 24.7 ± 4.7 kg/m^2^, indicating a generally normal weight range, and the mean age was 41.2 ± 16.3 years. Most of the included cases were women (18% men) (Table 1).

### 3.2. Feasibility and Reproducibility of sUMA CPP Measurements

Valid CPP measurements were obtained in all 106 participants included in the study. Considering a qualitative assessment, no reliability index is available in this case. The method’s feasibility was assessed, defined as the probability of obtaining valid measurements. In our analysis, the CPP sUMA evaluation demonstrated excellent feasibility, achieving a rate of 100%.

Intraobserver reliability was evaluated for the ViPLUS assessment. The intraclass correlation coefficient (ICC) was 0.905 (95% CI: 0.841 to 0.947) for the right thyroid lobe and 0.897 (95% CI: 0.839 to 0.942) for the left thyroid lobe, indicating good reliability for CPP sUMA measurement, confirming its reproducibility.

### 3.3. CPP sUMA Values in the Healthy Thyroid Cohort and the Influence of Subjects’ Characteristics

The findings for thyroid CPP sUMA measurements are summarized in Table 2.

**Figure 3:**
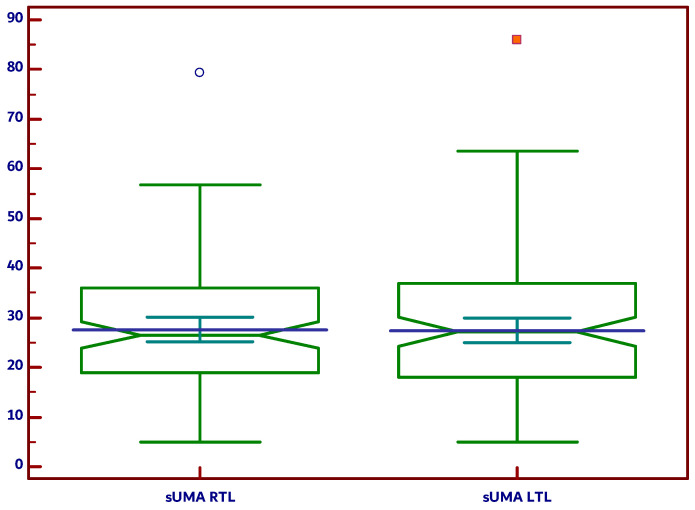
CPP sUMA values in the right thyroid lobe (RTL) and left thyroid lobe (LTL) (*p* = 0.8799); extreme values are represented as round blue circle and white fill for RTL and blue square with red fill for LTL.

Figure 3 demonstrates that there is no statistical difference (*p* = 0.8799) between measurements taken from the left and right lobes. Therefore, the mean of both values was used to represent the thyroid’s mean CPP sUMA or mean thyroid sUMA for subsequent analysis.

**Figure 4:**
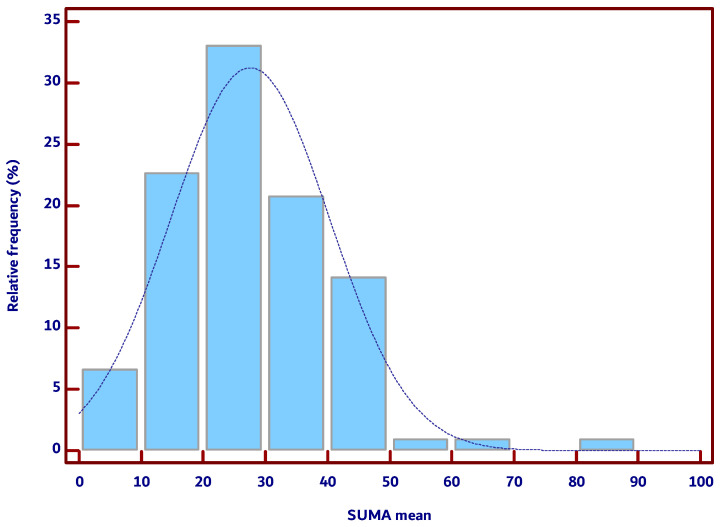
Relative frequency of mean thyroid CPP sUMA values; CPP—color pixel percentage; sUMA—Subtraction Ultra-Micro Angiography.

The mean values for normal thyroid sUMA mostly fell within the interquartile range (IQR) of 18.8–36.2%, as shown in Figure 4, which displays a histogram of the mean thyroid sUMA values’ distribution. Only one value significantly deviated from the overall trend, corresponding to the maximum values listed in Table 2.

In our analysis of the CPP sUMA values across different age subgroups (18–30 years, 30–50 years, and >50 years), we observed no statistically significant difference in mean CPP sUMA values (*p* = 0.497), as detailed in Table 3. This suggests that age did not have a significant impact on thyroid vascularity as measured by CPP sUMA in our cohort, indicating consistent microvascular patterns across these age subgroups.

Although the majority of patients in our group were female (82%), no significant differences in sUMA CPP values were observed between male and female participants (30.12 ± 5.86% in females vs. 31.53 ± 16.47% in males, *p* = 0.915).

Despite the fact that the mean sUMA CPP values were higher in patients of a normal weight (38 ± 18%) compared to those who were overweight (27 ± 11%), this difference did not reach statistical significance (*p* = 0.061). While the means suggest a potential trend, the *p*-value is slightly above the conventional threshold for significance. This implies that the observed differences may not be robust enough in this sample size, and further research with larger cohorts is needed to determine if this trend reflects a meaningful clinical difference.

In Table 4, the correlations between CPP sUMA measurements and various patient demographic, biochemical, and thyroid characteristics are presented. The data show no significant correlations between CPP sUMA and gender, age, TSH, FT4, depth, or thyroid volume, with all *p*-values above 0.05. However, there is a trend toward a negative correlation between CPP sUMA and BMI (r = −0.339, *p* = 0.083), suggesting that higher BMI may be associated with lower CPP sUMA values, though this result did not reach statistical significance.

## 4. Discussion

Color Doppler imaging is an ultrasound technique for evaluating vascular morphology. It was initially introduced to echocardiography in the early 1980s and has been increasingly applied in vascular diagnostics. CD uses a color map to depict blood flow direction and velocity overlaid on gray-scale images. Blood flow is color-coded based on direction and velocity, with flow moving away from the transducer shown in blue and toward the transducer shown in red. Lighter shades of these colors indicate higher velocities [18,19]. One limitation of the color Doppler method in evaluating small blood vessels is that the signal is received not only from the blood flow within the vessels but also from the vibrations of surrounding tissues. This overlap can reduce the accuracy of detecting and visualizing micro-vessels, as the low-speed blood flow in these vessels may be difficult to distinguish from the movement of adjacent tissues. Traditional Doppler techniques employ a one-dimensional wall filter design to eliminate clutter signal arising from tissue motion or transducer movement. While effective in suppressing low-frequency noise, this approach frequently leads to the unintentional removal of signals corresponding to low-velocity blood flow. Consequently, the accurate detection of low-velocity blood flow may be compromised [12,13].

The development of the advanced Doppler ultrasound technique was designed to visualize microvascular flow with high precision. Utilizing an intelligent algorithm that can eliminate clutter and signals from surrounding tissues, it can effectively distinguish low-speed blood flow signals from motion artifacts, enabling detailed assessment of micro-vessels and intricate vessel distributions that are often undetectable using conventional Doppler methods [18]. To achieve maximal sensitivity without introducing flash artifacts, careful optimization of ultrasound settings is necessary. This includes fine-tuning color gain and adjusting the width of the region of interest (ROI). Initial upregulation of color gain or reduction in ROI width may purposefully induce a flash artifact, which can then be minimized through gradual adjustments, ensuring clear visualization of microvascular structures [18].

Ultra-Micro Angiography technology introduces a novel approach to Doppler ultrasound by employing plane and divergent waves. This technique enables a faster sampling rate, significantly enhancing sensitivity in detecting microvascular flow. Additionally, a sophisticated wall-filtering algorithm is employed to accurately differentiate low-speed blood flow from low-speed tissue movement, thereby facilitating precise visualization of micro-vessels. The UMA feature includes three distinct sub-modes: cUMA, pUMA, and sUMA. Among these, sUMA stands out for its high spatial resolution and its capability to visualize micro-vessels in detail. This mode also allows the examiner to adjust background transparency across five grades (0–4). At grade 0, only vascular information is displayed, while grade 4 combines vascular and B-mode data, showcasing the brightest B-mode image. Notably, sUMA is the most sensitive sub-mode, capable of detecting even the smallest vessels with slow-velocity blood flow, making it an invaluable tool in assessing fine vascular structures. The evaluation of thyroid micro-perfusion can be quantitatively assessed using sUMA through automatic calculation of the color pixel percentage (CPP) index, the ratio between pixels representing the Doppler signal and the total pixels of the tissue of interest, offering an objective measure of the degree of vascularity. Establishing the value for normal thyroid parenchyma is the first step in verifying this novel technique [15].

To the best of our knowledge, this is the first study to investigate sUMA for thyroid perfusion. Our results show that in normal subjects, thyroid sUMA values range between 18.8% and 36.2%, with a mean value of 26.8%. As a result, sUMA values of approximately 27% can be interpreted as being representative of a normal thyroid. Superb Microvascular Imaging (SMI) is a similar, earlier-developed qualitative-only technique that revolutionized vascular imaging by eliminating background noise, providing a non-contrast alternative to Contrast-Enhanced Ultrasound (CEUS), which uses microbubble contrast agents to enhance blood flow visualization. With exceptional sensitivity for detecting slow blood flow in small vessels, SMI shows great promise for malignancy assessment [20].

So far, researchers have used UMA to obtain a high resolution for intestinal perfusion, both in healthy subjects and those with inflammatory bowel disease, and it shows potential for non-invasive treatment monitoring [16]. Another study conducted by Zhao et al. [21] investigated UMA to assess the activity of rheumatoid arthritis, with better detection of micro-vessels within the inflamed regions associated with high disease activity. Furthermore, UMA has been demonstrated to be an important diagnostic tool in lipedema, with excellent accuracy in the assessment of subcutaneous microvascularisation [22]. The applications of UMA can also be extended to other organs, like the breast and prostate [15].

sUMA technology also has several limitations: it is operator-dependent and the signal strength is influenced by depth and the amount of pressure applied to the probe, as the thyroid is a superficial organ. To enhance sensitivity for detecting micro-vessels and improve image resolution, the ROI should be positioned as superficially as possible. Increasing the depth of the ROI can reduce sensitivity due to the longer wait time required for returning echoes. Additionally, gentle pressure applied with the ultrasound probe is recommended to avoid collapsing the micro-vessels, thus preserving the visibility of fine vascular structures. In clinical practice, asking the patient to hold their breath can also be effective in minimizing motion artifacts, further enhancing the clarity of the microvascular imaging [12,13]. sUMA is a potentially valuable tool in pathological thyroid settings due to its ability to isolate microvascular blood flow from background tissue, providing high-resolution vascular detail. In diffuse diseases, such as autoimmune thyroiditis or Graves’ disease, sUMA can reveal increased or altered vascularity indicative of inflammation. For nodular disease, sUMA assists in differentiating benign from malignant nodules by highlighting abnormal blood flow patterns, supporting early and accurate diagnosis. This makes sUMA a powerful tool for detailed vascular assessment in both diffuse and nodular thyroid pathologies.

Future research should focus on validating these findings through multicenter studies, assessing sUMA’s role in disease progression and treatment response and comparing its diagnostic accuracy against established imaging modalities.

The integration of sUMA into thyroid imaging has the potential to significantly improve patient outcomes by enabling earlier and more accurate diagnoses. By detecting subtle microvascular changes, sUMA may aid in the early identification of thyroid pathologies, allowing for timely intervention in conditions such as autoimmune thyroiditis or malignancies. Additionally, its ability to provide quantitative vascular data enhances risk stratification, helping to distinguish between benign and malignant nodules more effectively, which may reduce unnecessary biopsies and overtreatment. In diffuse thyroid diseases like Graves’ disease or chronic thyroiditis, sUMA could serve as a valuable tool for monitoring vascular alterations over time, offering a non-invasive approach to assessing disease progression and treatment response. Ultimately, the improved vascular detail provided by sUMA may enhance clinical decision-making, contributing to more personalized and effective patient management.

## 5. Conclusions

sUMA is a revolutionary Doppler technique that is reproducible and feasible for the evaluation of thyroid parenchyma. The results of our research demonstrate that this innovative method plays a crucial role in the quantitative assessment of thyroid microperfusion, paving the way for future evaluations of thyroid pathology.

## Figures and Tables

**Table 1:** Baseline characteristics of study participants.

Variable	Distribution	Summary Statistics	Values
Age (years)	Normal	Mean ± SD	41.2 ± 16.3
BMI (kg/m^2^)	Normal	Mean ± SD	24.7 ± 4.7
Thyroid Volume (mL)	Normal	Mean ± SD	12.3 ± 4.2
TSH (µIU/mL)	Normal	Mean ± SD	2.3 ± 1.6
Free T4 (pmol/L)	Normal	Mean ± SD	15.5 ± 2.5
ATPO (IU/L)	Non-normal	Median (IQR)	18 (12.5–27)
ATG (IU/L)	Non-normal	Median (IQR)	1.7 (0.9–3.1)
Gender (Male/Female)	Qualitative	Percentages	19 M/87 F (18% M/82% F)

BMI—body mass index (kg/m^2^); SD—standard deviation; TSH—thyroid-stimulating hormone; ATPO—anti-peroxidase antibodies; ATG—antithyroglobulin antibodies; IQR—interquartile range; M—male; F—female.

**Table 2:** Power Doppler ultrasound-based CPP sUMA measurements of thyroid tissue in healthy participants.

Parameter			Value
sUMA CPP (%)	Right Thyroid Lobe	Median (IQR)	26.5 (18.8–36)
		Min	5
		Max	79.2
	Left Thyroid Lobe	Median (IQR)	27.1 (18–37)
		Min	5
		Max	86
	Mean Thyroid	Median (IQR)	26.8 (18.8–36.2)
		Min	5.75
		Max	82.6
Depth		Median (IQR)	1.8 (1.6–1.8)

sUMA—Subtraction Ultra-Micro Angiography; CPP—color pixel percentage; IQR—interquartile range.

**Table 3:** Comparison of CPP sUMA values across age subgroups.

Age Subgroup	CPP sUMA (%)	*p*
18–30	30 ± 15	0.497
30–50	35 ± 21	
>50	32 ± 13	

CPP—color pixel percentage; sUMA—Subtraction Ultra-Micro Angiography.

**Table 4:** Correlations between CPP sUMA measurements and patient demographic, biochemical, or thyroid measurement characteristics.

		BMI	Gender	Age	TSH	FT4	Depth	Thyroid Volume
CPP sUMA mean	r *p*	−0.339 0.083	−0.025 0.884	−0.045 0.817	0.225 0.385	0.274 0.303	−0.018 0.857	−0.065 0.506

r-Pearson correlation coefficient; CPP—color pixel percentage; sUMA—Subtraction Ultra-Micro Angiography.

## Data Availability

The original contributions presented in the study are included in the article, further inquiries can be directed to the corresponding author.
